# Optimizing forest structure for sustainability: a review of structure-based management effects on stand quality

**DOI:** 10.48130/forres-0025-0024

**Published:** 2025-10-29

**Authors:** Qiming Liao, Quan Qiu, Jie Gao, Qiang Liu, Qin Su, Yue Yang, Peilin Xie, Yutian Xin, Xiaolong Zhao, Pan Wan

**Affiliations:** 1 College of Forestry, Northwest A & F University, Yangling 712100, China; 2 Guangdong Key Laboratory for Innovative Development and Utilization of Forest Plant Germplasm, College of Forestry and Landscape Architecture, South China Agricultural University, Guangzhou 510642, China; 3 Key Laboratory for the Conservation and Regulation Biology of Species in Special Environments, College of life science, Xinjiang Normal University, Urumqi 830054, China; 4 College of Forestry, Hebei Agricultural University, Baoding 071000, China; 5 College of Ecology, Lanzhou University, Lanzhou 730000, China

**Keywords:** Structure-based forest management, Stand quality, Stand productivity, Stand structure, Soil-plant interaction, Forest ecosystem, Sustainable forestry

## Abstract

Structure-based forest management (SBFM) has emerged as an innovative silvicultural approach that optimizes the spatial arrangement of trees to emulate natural forest structures and promote sustainability. Despite increasing applications of SBFM, a comprehensive synthesis of its impacts on stand quality and the underlying mechanisms remains lacking. This review synthesizes 126 peer-reviewed studies (2007–2025) to evaluate the multidimensional effects of SBFM on forest stand growth, structure, soil properties, and stability. Evidence indicates that SBFM enhances tree growth by reducing competitive pressure, improves diameter distribution and species mingling, thereby approximating natural stand patterns, and enriches soil carbon and nutrient pools through increased litter input and microbial activity. These structural adjustments collectively foster a positive feedback loop that integrates aboveground productivity, belowground processes, and overall ecosystem resilience. Future research should prioritize cross-regional ecological monitoring, mechanistic experiments that link structural optimization to biodiversity and carbon sequestration, the integration of artificial intelligence (AI) and remote sensing for precision management, and improvements in forest stand quality evaluation systems. Overall, SBFM markedly improves stand quality and constitutes a promising strategy for sustainable forest management that enhances ecological resilience.

## Introduction

Natural forests are the cornerstone of terrestrial ecosystems, providing indispensable services such as carbon sequestration, biodiversity maintenance, and climate regulation^[1]^. Their complex structures, shaped by natural disturbances (e.g., wildfires, windthrow, and insect outbreaks), confer resilience and sustain biogeochemical cycles over millennia^[2]^. However, widespread deforestation and degradation, exacerbated by intensified anthropogenic disturbances and climate extremes, have resulted in alarming declines in forest quality and ecosystem function worldwide. These trends threaten global carbon neutrality targets and biodiversity conservation goals^[3,4]^. Consequently, there is an urgent need for management approaches that not only restore forest functions and resources but also emulate the structural complexity of natural forests to enhance resilience. However, conventional silvicultural systems often rely on coarse stand-level indicators and fail to explicitly account for fine-scale spatial patterns. This limitation reduces their capacity to optimize competitive interactions and promote structural heterogeneity. In response, structure-based forest management (SBFM) has emerged as an innovative framework that quantifies the spatial relationships among neighboring trees and guides targeted interventions aimed at approximating a natural forest structure and improving stand quality^[5−8]^.

**Figure 1:**
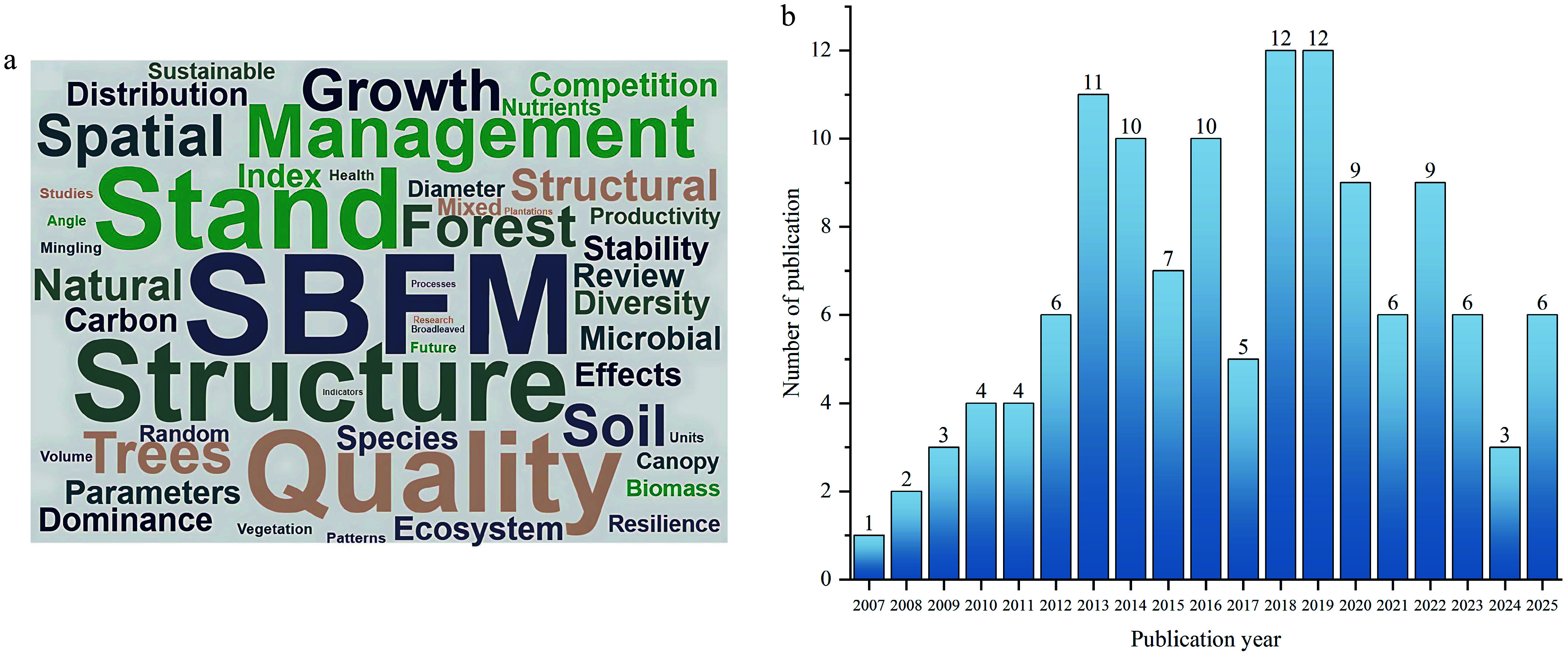
Research hotspots and publication trends in structure-based forest management (SBFM). The word cloud shows the most frequent keywords from 126 studies (2007–2025), highlighting major themes such as stand structure, soil quality, and productivity (a). The line chart indicates a steady rise in SBFM publications, reflecting growing global interest in its application to sustainable forest management (b).

Rooted in systems theory, SBFM posits that an ecosystem's function is determined by its structure. The management approach therefore centers on the five-tree structural unit, using quantitative spatial parameters to evaluate a stand's spatial distribution, species composition, size dominance, and competition intensity^[9,10]^. This diagnostic process enables targeted interventions, such as selective thinning, to steer the stand's structure toward a more random distribution and the higher heterogeneity characteristic of natural forests^[11]^. Widely adopted worldwide, SBFM has significantly improved tree growth rates, diameter distribution, species mingling, and stand-level productivity and stability^[12−14]^. Moreover, by promoting diverse spatial configurations, SBFM facilitates light penetration, nutrient redistribution, and understory regeneration, thereby indirectly enhancing soil carbon and nutrient pools. Fig. 1 illustrates the number of SBFM publications in recent years and the core research areas. Nevertheless, existing research often focuses on isolated outcomes (e.g., diameter growth, basal area increment, or short-term changes in spatial indices) without integrating above- and below-ground processes into a holistic assessment of stand quality. Long-term (> 10 years) effects of SBFM on soil microbial communities, nutrient cycling, and ecosystem resilience remain largely unexplored. Addressing these gaps is essential for developing mechanistically informed silvicultural strategies that strengthen the adaptive capacity of forest ecosystems under accelerating global change.

Forest quality is a comprehensive metric for evaluating the overall condition and value of forests, and it plays a vital role in forest protection and resource management^[15]^. Silviculture is inherently dynamic, requiring management objectives to continually adapt to changing societal demands and global environmental shifts while sustaining or enhancing the ecosystem's adaptive capacity^[16]^. Therefore, assessing forest quality is crucial for optimizing wood production^[17]^, safeguarding biodiversity^[18]^, forecasting carbon sequestration potential^[19]^, and guiding silviculture planning^[16]^. Forest quality can be evaluated at two complementary levels: Landscape and stand. The landscape-level assessment emphasizes the ecological, social, and economic significance of forests, reflecting their broad environmental contributions^[20−22]^. In contrast, stand-level assessments capture the intrinsic condition of localized stands, which constitutes the primary focus of this review and is typically quantified through field-based metrics, including tree size, canopy characteristics, vegetation cover, species diversity, biomass stocks, and soil indicators^[23−25]^. Stand quality assessments integrate diverse biotic and abiotic factors, with methods tailored to specific management objectives^[26]^. Building on this foundation, we propose an SBFM-aligned framework emphasizing five key elements (i.e., productivity, structure, health, diversity, and regeneration) and highlight the structural dimension through use of eight spatial and nonspatial indicators.

**Figure 2:**
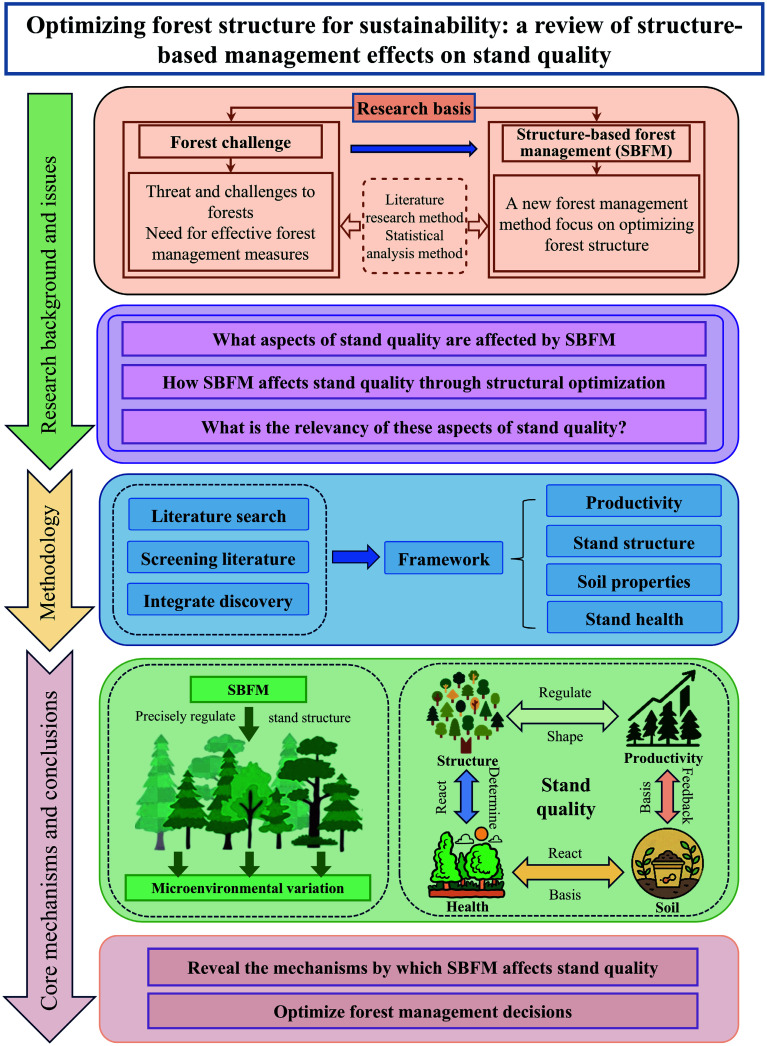
The diagram summarizes the analytical framework linking SBFM practices with four interrelated components (stand productivity, structure, soil, and health), illustrating how spatial optimization enhances forest quality through feedback between above- and below-ground processes.

SBFM has proven effective in enhancing stand productivity, optimizing spatial distribution, improving soil health, and advancing overall stand condition across diverse forest ecosystems, yet most studies still examine individual variables (e.g., soil properties or spatial indices) rather than providing an integrated assessment of its multifaceted effects. Moreover, significant challenges remain, including the scarcity of long-term (> 10 years) monitoring to capture successional dynamics, and the limited geographical representativeness of current research, which restricts the generalizability of findings. To bridge these gaps, this review systematically synthesizes the impacts of SBFM on four core dimensions of stand quality: (1) Stand productivity (i.e., diameter at breast height, volume, biomass, and carbon storage), (2) stand structure (i.e., spatial and nonspatial attributes), (3) soil (i.e., carbon and nutrient cycling, microbial and enzymatic responses), and (4) stand health. Emphasis is placed on structural attributes, as these are the central mechanism through which SBFM influences the ecosystem's function. The relationships among these four aspects of stand quality and their mechanisms of action in influencing stand development were investigated, representing the novelty of this paper. Fig. 2 illustrates the conceptual framework of this review and its analytical approach. Finally, key knowledge gaps are identified and future research priorities outlined, including long-term cross-regional monitoring, mechanistic experiments linking structural optimization to biodiversity and carbon sequestration, and the use of artificial intelligence (AI) and remote sensing for precision management. Collectively, this review provides a multidimensional and mechanistic understanding of how SBFM improves stand productivity, structure, soil conditions, and ecosystem resilience, offering actionable insights for adaptive forest management aimed at enhancing forest quality.

## Methodology

This review was conducted in in accordance with the Preferred Reporting Items for Systematic Reviews and Meta-Analyses (PRISMA) framework to ensure methodological rigor, transparency, and replicability throughout its analytical design, literature screening, and synthesis of evidence^[27]^. A comprehensive search strategy was implemented without regional, linguistic, or publication type restrictions. The search covered five multidisciplinary databases including Web of Science (WoS), Scopus, SpringerLink, China National Knowledge Infrastructure (CNKI), and Wanfang Database to identify peer-reviewed literature published between January 2007 and October 2025. Search terms included conceptually related phrases such as "structure-based forest management", "stand quality", "tree growth", "stand structure", "stand stability", "soil quality", and "sustainable forestry", combined using Boolean operators.

**Figure 3:**
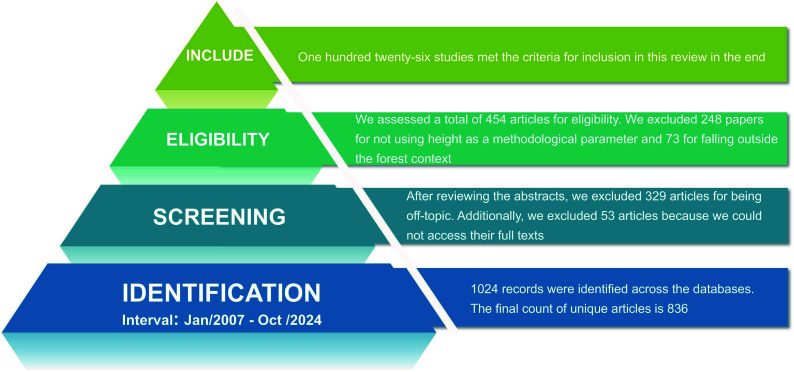
PRISMA-compliant literature screening process for the systematic review.

**Figure 4:**
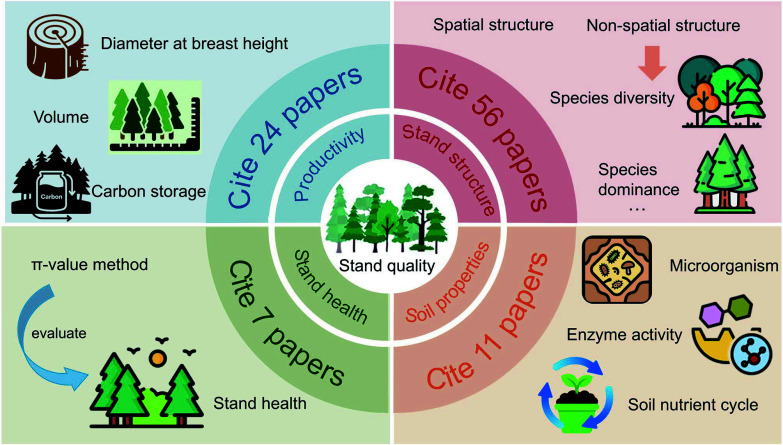
The number of references cited for each aspect of forest stand quality in the review. The π-value method, used for assessing stand health, standardizes the evaluation by calculating the ratio of observed to ideal optimal states

Publications were filtered on the basis of publication year, language (Chinese/English), and document type (peer-reviewed articles). Bibliographic records were exported in RIS format and deduplicated using EndNote^[28]^, yielding an initial pool of 836 articles. A subsequent methodological evaluation further refined the selection. Studies were included if they had (1) an empirical evaluation of SBFM (or analogous spatial optimization methods) in natural/plantation forests, (2) a quantitative assessment of ≥ 2 stand quality dimensions (growth, structure, stability, or soil properties) using pre-post management data or control comparison, and (3) standardized protocols for data collection and statistical analysis. Similarly, the exclusion criteria ruled out (1) nonempirical studies lacking field validation, (2) research unrelated to SBFM-stand quality relationships, and (3) datasets with insufficient replication (< 3 samples), incomplete metrics, or methodological flaws (e.g., nonstandard measurements). After sequential screening, 126 articles qualified for systematic synthesis (Fig. 3). We additionally illustrate the reference distribution across the paper's sections, clarifying the organizational structure of this review (Fig. 4).

A four-domain analytical matrix was developed to standardize the content analysis (Supplementary Table S1). The first domain, "general information", records key bibliographic information such as author, journal, and publication year. The second ("methodology") summarizes the research methods, including study areas, temporal scales, and analytical approaches. The third domain, "key indicators", lists evaluation metrics related to stand quality. Finally, the "discussion" domain captures the major findings, scientific contribution, limitations, and proposed directions for future research. All extracted data were cataloged in structured Microsoft Excel templates to enable cross-study comparisons, maintain analytical consistency, and enhance transparency. This methodological framework provides the basis for a systematic evaluation of SBFM's impacts on stand growth, structure, soil properties, and stability.

### Structure-based forest management

**Figure 5:**
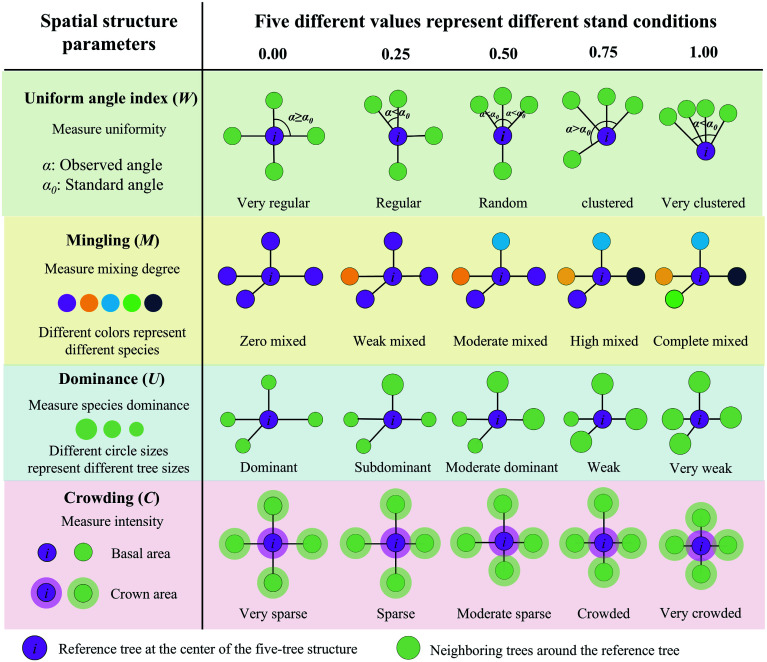
Schematic diagram of spatial structure parameters based on five-tree structural units. This figure illustrates four key parameters for quantifying stand spatial structure: (1) The uniform angle index (*W*), where *W *= 0.5 represents an ideal random distribution typical of natural forests; (2) mingling (*M*), where *M *= 1 indicates the greatest level of species segregation; (3) dominance (*U), where U *= 0 identifies trees with significant competitive dominance; and (4) crowding (*C*), where *C *= 1 represents the strongest competitive stress. Through these four parameters, structure-based forest management can achieve precise diagnoses of stand spatial structure, thereby regulating it to an ideal state.

**Figure 6:**
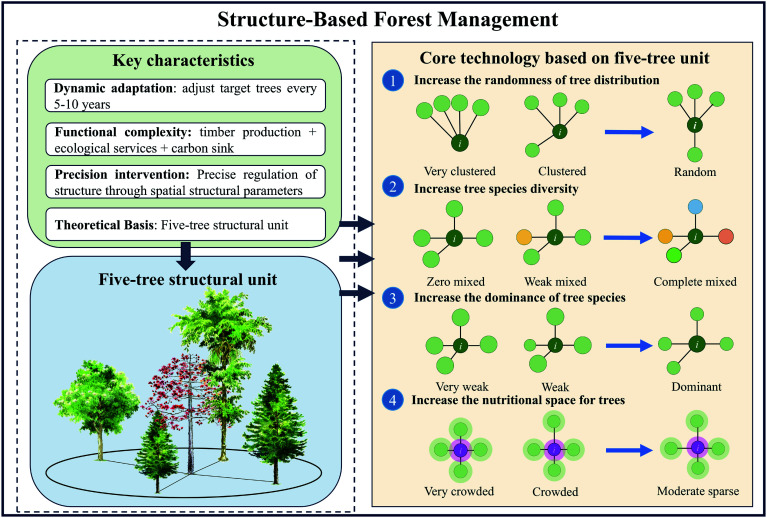
Schematic diagram illustrating structure-based forest management (SBFM). The five-tree structural unit consists of a central tree and the four nearest trees within its unit cell. Based on the "five-tree structural unit," stand structure can be precisely regulated and optimized through spatial structural parameters, guiding forest development toward natural forests characteristics such as random distribution, complete species mixed, dominant tree species, and moderate sparse spacing.

The primary objective of forest management is to optimize forest structure through targeted adjustments, enhancing trees' competition dynamics and overall forest quality^[8]^. Systems theory posits that the structure of a system determines its function. Building on this principle, Hui et al. developed SBFM, an innovative approach that optimizes the spatial relationships between a tree and its four nearest neighbors^[9,12]^. Originating from Germany's close-to-nature forest management philosophy, SBFM emphasizes tree-oriented silviculture as its core principle, with ecology taking priority^[11]^. Its core methodology refines the stand's spatial structure through four stand spatial structure parameters (SSSPs) (Fig. 5), creating more randomly distributed trees (or random structural units) around the target trees. This optimization enhances structural diversity, adjusts species composition through selective thinning, and fosters stable forest development^[13,14,29]^. To facilitate understanding of SBFM, a concept diagram was used to illustrate the core features, theoretical basis (the five-tree structure), and key technologies (Fig. 6).

Stand structure is defined as the architectural and functional components governing a forest's physical organization^[30]^. It is a key indicator of forest conditions, and helps track how a stand grows and changes over time^[31]^. SBFM quantifies stand structure using units formed by a reference tree and its four nearest neighbors^[32,33]^ and thus has resulted in four key spatial parameters (Fig. 5). (1) The uniform angle index (*W*) measures the uniformity of spatial distribution, where *W* = 0.5 indicates an ideal random distribution characteristic of natural forests^[34]^. (2) Mingling (*M*) quantifies species diversity and segregation, wheer *M* = 1 indicates the greatest species segregation^[35]^. (3) Dominance (*U*) assesses the size of the reference tree relative to its neighbors, where *U* = 0 identifies the trees with significant competitive dominance^[36]^. (4) Crowding (*C*) evaluates canopy density and the intensity of resource competition, where *C* = 1 represents the strongest competitive stress; usually, the optimal thresholds vary by forest type^[37]^. These four structural parameters collectively describe the position and condition of an individual tree within a community by analyzing the neighboring trees' distribution, species composition, size dominance, and resource competition intensity. Each parameter uses discrete values (0.00, 0.25, 0.50, 0.75, 1.00) that represent stand conditions and allow precise ecological interpretations. The standardized mean of four parameters is used to calculate the stand's spatial structure index (FSS) on the basis of the unit circle method and the π value rule^[38]^.

SBFM uses large sample plots (≥ 2,500 m^2^) to collect forest data with the help of station instruments combined with quadrat and point sampling techniques^[9,12]^. In point sampling, a minimum of 49 survey points are established to calculate structural parameters for the four nearest trees at each point, ensuring accurate characterization. Key management interventions include: (1) adjusting the spatial distribution by optimizing the uniform angle index so each target tree is surrounded by four randomly positioned neighbors; (2) enhancing species composition by amplifying the mingling index to promote biodiversity and species segregation within the microhabitat of target tree; (3) regulating the competitive hierarchy by balancing interspecific competition using the dominance index for optimizing light availability from one or two directions; and (4) optimizing resource allocation by balancing crowding indices to secure nutrient availability for individual trees. As a data-driven approach, SBFM quantifies stand structure, clarifies biodiversity patterns, and identifies linkages between spatial configuration and tree competition. By integrating empirical measurements with analytical models, it supports precision management strategies for quantitative structural adjustments and optimization^[10]^.

### Impact on stand productivity

#### Stand diameter at breast height

Diameter at breast height (DBH) serves as a foundational metric in forest surveys, providing critical insights into tree size, growth dynamics, age estimation, and biomass calculation^[39]^. Changes in DBH reflects the effects of forest management practices on trees' growth and the stand's structural dynamics, forming the basis for informed management decisions^[40]^.

Research indicates that SBFM significantly enhances stand-level and DBH growth, approaching the productivity of prime natural forests^[41,42]^. This improvement is attributed to SBFM's selective removal of small, unhealthy, or underperforming trees, which reduces competition for resources and promotes growth in remaining trees^[43]^. For instance, Wan^[44]^ evaluated the impacts of forest management on a natural *Quercus aliena* var*. acutiserrata* forest, and reported that the average annual growth rate of stand DBH was highest under SBFM (2.13%) compared with unmanaged controls, indicating the positive effects of SBFM. However, Chen et al.^[45]^ found higher diameter growth in unmanaged stands, likely caused by natural mortality reducing stand density and inflating the mean diameter values. Notably, substantial methodological differences, such as the time of operation of SBFM, and the area of operation in the research forest stands, limiting comparability across these studies.

Overall, these findings show that SBFM enhances growth while maintaining the ecosystem's integrity through targeted structural interventions that reduce competition and support sustainable stand development. However, limited sample sizes and short monitoring periods constrain the generalization of results across species or regions.

#### Stand volume

Stand volume, representing the total wood volume within a forest stand (typically measured in m^3^ and considered to be a critical forest management metric for quantifying the available timber and biomass^[46]^). Monitoring its changes over time is essential for sustainable management, helping to detect shifts in stand productivity and to guide conservation strategies^[47]^.

Studies demonstrate that SBFM effectively enhances both the average annual growth rate and stand volume. For example, Chen et al.^[45]^ conducted an in-depth analysis of SBFM's effects on the quality of broadleaf Korean pine forests located in northeastern China. They reported that managed stands exhibited 0.5 m^3^ greater average annual volume growth and 0.22% higher growth rates than the controls. These findings confirm that SBFM is effective in improving the average annual growth and the growth rate of stand volume^[41,48]^. This may be because, after SBFM optimizes the stand's structure, more nutrients become available for absorption by tree roots, thereby promoting treea' growth and reducing mortality^[49,50]^.

Notably, the initial stand volumes may temporarily decline following selective removal of diseased, decayed, or structurally unsound trees after SBFM. While reducing short-term wood stocks, this intervention mitigates resource competition. Longitudinal data confirm that SBFM-managed stands consistently surpass the controls in annual growth rates within 3–5 years of implementation^[45]^, underscoring the dual capacity of SBFM to balance immediate resource use with long-term ecosystem vitality. However, there is currently limited research evaluating the longer-term effects of SBFM, such as those lasting over 10 years.

#### Biomass and carbon storage

Stand biomass refers to the total mass of living organic matter within a stand^[51]^. In forest management, this metric is essential for estimating the potential timber yield and for planning sustainable harvesting^[52,53]^. Numerous studies have demonstrated that SBFM significantly enhances stand biomass^[45,50,54]^ through (1) optimization of the spatial structure^[55]^, enabling sunlight penetration for growth or (2) improvements in soil health^[56]^, which improves soil bacterias' diversity and abundance for carbon and nutrient cycling via improved respiration and assimilation processes^[57]^. An analysis of annual growth rates confirms that SBFM enhances long-term biomass accumulation despite short-term reductions^[44]^.

Forests and other biomass-rich ecosystems function as major carbon sinks. In forest systems, plants assimilate atmospheric CO_2_ through photosynthesis, converting it into organic matter that stores carbon in plant tissues and contributes to biomass accumulation^[58,59]^. This indicates the intrinsic link between biomass and carbon storage, as biomass serves as a primary carbon reservoir. Zhang et al.^[60]^ reported that aboveground carbon storage in stands subjected to SBFM remained lower than in unmanaged stands over an extended period. This transient reduction may result from altered resource competition dynamics following thinning^[61,62]^, which can temporarily diminish photosynthetic efficiency and carbon assimilation capacity. Moreover, soil carbon storage is modulated by aboveground biomass through the input of litter and root exudates^[63]^. These interconnected mechanisms highlight how forest ecosystems function as cohesive units, with all components interacting to govern carbon cycling.

### Impact on stand structure

Stand structure, defined by the spatial and functional arrangement of trees within a forest^[64]^, reflects key ecological processes such as regeneration, competition, and self-thinning, which are essential for understanding ecosystem function^[65−68]^. It serves both as a critical component in forest analyses and silvicultural management^[69,70]^. This review assesses the impacts of SBFM on forest stands across both spatial and nonspatial structures.

#### Stand density

Stand density measures how many trees grow in a given area and how strongly they compete^[71]^, showing trees' abundance and competition intensity. It strongly affect the forest's growth, productivity, and health by shaping resource use, canopy structure, and ecosystem resilience^[72]^. Chen et al.^[45]^ examined the effects of SBFM on stand density in broadleaf red pine forests in Northeast China. Their findings revealed that during the initial post-intervention phase, SBFM-managed stands showed 0.2 m^2^ higher mean annual basal area increment (BAI) and a 0.9% greater annual growth rate compared with the unmanaged controls, and these stands maintained a 0.12 m^2^ BAI advantage and a 0.5% faster growth rate during subsequent monitoring. However, later on, Chen^[43]^ observed that SBFM reduced stand density through selective harvesting in the early stage and natural mortality in later stages. This demonstrates SBFM's capacity to enhance both basal area expansion and volumetric growth, thereby boosting stand productivity.

#### Stand diameter distribution

Stand diameter distribution refers to the size of trees, measured as DBH, and their abundance in a forest area, and is a key indicator for describing a stand's structure and growth^[73]^. Chen et al.^[45]^ found a significant post-SBFM shift toward medium- and large-diameter trees owing to the selective removal of small-diameter trees. The removal of structurally unhealthy and small trees reduces competition and accelerates the growth and dominance of medium and large cohorts. Chen^[43]^ further confirmed these structural changes through diameter distribution curve analysis, showing that SBFM reduced the proportion of small-diameter trees, increased the proportion of medium- and large-diameter trees, and enhanced the dispersion of differences in stand diameter. Zhao et al.^[74]^ applied SBFM in broadleaf Korean pine mixed forest, and reported that SBFM can maintain the reverse "J"-shaped distribution characteristic of natural forests. These findings highlight SBFM's effectiveness in balancing silvicultural objectives with ecological integrity by harnessing natural ecosystem dynamics.

#### Species dominance

Species dominance is defined as the relative importance of a tree species within a forest community; is based on abundance, frequency, or ecological function; and is critical for understanding a forest's structure, function, and dynamics^[75,76]^. Research demonstrates that SBFM effectively enhances target species' dominance by accelerating their growth for sustaining dominance over long-term^[42,74]^, which is similar to the characteristics in the top forest community. This outcome of SBFM's strategic structural adjustments (e.g., selective thinning) and optimized species distribution reduces competitive pressure and improves resource availability for the target species^[43,74]^. Furthermore, SBFM restores and maintains keystone species' dominance^[43]^, enabling them to maximize their ecological functions (e.g., carbon sequestration, habitat provision) and bolster the ecosystem's resilience. However, shifts in species dominance usually occur gradually and remain relatively stable over time, reflecting the slow pace of natural succession^[45]^.

#### Species diversity

Species diversity quantifies the variety of species within an ecosystem or habitat, encompassing species richness, composition, and distribution^[77]^. It serves as a critical indicator of community functionality. Ecological diversity is typically measured using Simpson and Shannon diversity indices, with cross-system comparisons using the Sorensen coefficient^[78]^. Empirical studies demonstrated that SBFM enhances species diversity. Research indicates that SBFM resulted in significantly higher herbaceous species richness and Shannon–Wiener diversity in the managed stands compared with the unmanaged controls^[41,79]^. Zhang et al.^[80]^ also obtained a 47.7% increase in understory herbaceous diversity following optimization of stand spatial structure. However, the existing evidence is largely limited to herbaceous layers, and the effects on woody species and overall biodiversity remain insufficiently studied.

Mechanistically, SBFM's selective thinning reduces stand density, increasing light penetration and airflow. This creates open canopy structures, enhanced habitat heterogeneity, and expanded ecological niches. These microhabitat changes improve the availability of resources such as light and water, and create more ecological niches, facilitating understory plant recruitment and regeneration. Collectively, these processes foster species' coexistence and enhance biodiversity.

#### Canopy structure

The forest canopy's structure serves as the primary interface for matter and energy exchange between forests and the atmosphere^[81−83]^. Management practices like SBFM influence canopy structure by modifying tree species composition, stand density, and spatial distribution^[83−85]^, thereby regulating ecological processes, including light interception and gas exchange^[86,87]^. A key metric for assessing these changes is the leaf area index (LAI), which quantifies the canopy's density and functional capacity. Wan et al.^[84]^ demonstrated that SBFM increases stand LAI, with significantly reduced mean leaf angles, reflecting enhanced light-use efficiency. Stand productivity is strongly and positively correlated with LAI^[88,89]^, so these structural adjustments may have significant effects on productivity. Furthermore, SBFM modulates canopy openness to enhance understory light availability. Reduced leaf angles suggest a more horizontal leaf orientation, which improves the penetration and distribution of internal light. Feng et al.^[90]^ also reported that SBFM enhances direct radiation interception by the upper canopy layers, which mitigates light competition and fostering favorable understory conditions for plant recruitment and growth. Collectively, these findings highlight SBFM's capacity to optimize canopy structure, elevate light-use efficiency, and improve the ecosystem's productivity on a sustainable basis.

### Stand spatial structure

A system's structure fundamentally determines its functionality, with rationally organized systems exhibiting superior performance. This principle holds profound relevance for forest ecosystems, where a stand's spatial structure governs its developmental trajectories and ecological functions^[29,91]^. Specifically, spatial structure shapes competitive interactions and niche partitioning among trees, directly influencing a stand's vitality through its health, growth potential, and stability^[92]^. Optimizing the spatial structure can enhance a stand's resilience and productivity^[59]^. Analysis of stand spatial structure relies on four key parameters, namely the uniform angle index (*W*), mingling (*M*), dominance (*U*) and crowding (*C*)^[11, 93]^. These parameters provide a comprehensive framework for understanding the spatial relationships within stands and their management implications.

#### Univariate distribution of spatial structural parameters

Univariate distribution analysis quantifies the distribution frequency of trees exhibiting specific structural characteristics within stands. This method is based on structural units consisting of a central reference tree and its four nearest neighbors, and analyzes the frequency distributions of five possible values for each spatial parameter. This parametric characterization enables researchers to systematically identify and interpret subtle structural patterns within a stand, thereby revealing distinct features of stand structure relative to a given aspect^[13]^.

Wan et al.^[55]^ found a clustered distribution before the implementation of SBFM in a natural *Quercus aliena* var. *acutiserrata* forest. Following the SBFM interventions, the trees' distribution shifted to random spatial patterns, widely regarded as optimal for stand structures, thereby demonstrating SBFM's efficacy in aligning managed forests with natural spatial distributions^[94]^. Furthermore, SBFM significantly enhances species mixing within a stand compared with other methods with minimal mixing impacts^[48,95]^. This improvement stems from SBFM's ability to reduce conspecific density around the subject trees and create establishment niches for other species. The degree of mixing in a stand reflects species diversity within a forest, with higher values showing greater correlations with biodiversity. Previous research has demonstrated that stands with higher proportions of mixed species exhibit more optimal spatial structures^[94,96]^. Additionally, highly mixed forests contain a greater proportion of dominant tree species, indicating that large trees tend to lead to higher interspecific mixing than smaller trees^[97−99]^. Collectively, these findings suggest that the increased mixing facilitated by SBFM creates favorable conditions for tree growth and stand development.

#### Bivariate distribution of spatial structural parameters

Bivariate distribution analysis examines the joint occurrence of two structural parameters^[100]^. This facilitates assessments of species mingling among trees of varying dominance levels, spatial patterns of trees with distinct mingling attributes, and the arrangement of trees exhibiting different dominance hierarchies. By integrating two structural parameters derived from neighboring tree relationships, this method simultaneously captures two aspects of stand spatial structure, revealing microstructural attributes and comprehensively reflecting structural complexity^[29,100]^. Similarly, optimal stand quality is defined by three key attributes including high species diversity, a substantial proportion of dominant trees, and a spatially random tree distribution^[^^99^^−^^101^^]^. These form a bivariate framework identifying three advantageous microstructures in mature natural forests^[12]^: (1) Randomly distributed trees with high species diversity, (2) randomly distributed dominant trees, and (3) dominant trees exhibiting high species mixing. Favorable microstructures promote resource use efficiency, maintain competitive balance, and support ecological stability. In contrast, unfavorable microstructures are characterized by low species mixing, nonrandom spatial patterns, and a dominance of suppressed or inferior trees. These conditions are often correlated with reduced stand productivity, impaired nutrient cycling, and increased vulnerability to disturbances. Therefore, forest management practices aim to minimize such unfavorable microstructures to better emulate natural stand dynamics.

Empirical evidence shows that SBFM uniquely achieves immediate, sustained reductions in disadvantageous microstructures. Wan et al.^[55]^ reported significantly lower proportions of disadvantageous microstructures that were maintained even four years post-intervention, alongside rapid increases in advantageous structures. However, Chen et al.^[45]^ observed no short-term improvements in advantageous microstructures, as the stands under SBFM retained higher advantageous and lower disadvantageous microstructure proportions than the controls over time. Collectively, these findings demonstrate SBFM's effectiveness in optimizing forest spatial structure by enhancing ecologically beneficial microstructures and reducing unfavorable ones, leading toward sustainable silviculture.

#### Tree competition

Competition is a fundamental interaction that plays a pivotal role in a forest stand's development^[101]^. Tree competition is defined as the antagonistic relationships among neighboring trees as they concurrently struggle to access finite resources, including light, soil nutrients, water, and spatial area^[102]^. These competitive dynamics directly govern individual trees' growth and survival, and stand structure^[103]^.

Quantitative indices like the structure-based competition index (SCI)^[104]^, basal area in larger trees (BAL)^[105]^, and competition index (CI)^[106]^ effectively quantify the intensity of competition, revealing the impacts of resource limitation on trees' performance. Chen^[43]^ analyzed SCI and BAL across managed and unmanaged stands over multiple growth periods, and reported that SBFM mitigates competition pressure. Similarly, Jiang et al.^[48]^ measured a 51.4% reduction in CI values in secondary forests of *Phoebe zhennan* after SBFM. Therefore, it was observed that SBFM not only preserved natural successional processes but also fostered stable forest development.

### Impact on soil

The forest soil composition dynamically responds to aboveground vegetation and environmental factors (e.g., nutrient availability, temperature, moisture), and is modulated by management practices like SBFM^[107,108]^. Storing more than 40% of terrestrial ecosystem carbon^[109]^, soil plays a key role in global carbon cycling.

Ou et al.^[110]^ analyzed soil organic carbon (SOC), total nitrogen (TN), and total phosphorus (TP) in the top 30 cm of secondary oak forests in Xiaolongshan, Gansu Province after 8 years of SBFM treatment. They observed significant increases in SOC storage (+ 13.75%), TN (+ 0.24%), and TP (+ 37.30%) compared with unmanaged stands. Such nutrient accumulation results largely from SBFM-induced structural optimization, including enhanced species mingling, a larger share of large-diameter trees, and increased biomass. These improvements, in turn, stimulate litter production and nutrient return, while accelerating litter decomposition and the subsequent release of phosphorus^[49]^. Wan et al.^[56]^ reported no significant changes in SOC in a natural oak forest under SBFM, attributed to minimal differences in litter biomass, fine-root dynamics, and carbon- or nitrogen-cycling enzyme activities. These divergent outcomes highlight the sensitivity of soil to environmental changes and are potentially influenced by forest management as well as the soil type and management duration.

Management practices like SBFM induce structural and functional modifications in above- and belowground compartments. These interventions alter understory vegetation patterns, modify the soil, and influence microclimatic conditions, ultimately affecting soil microorganisms or certain enzymes' activity^[5,107,111]^. Soil microbial biomass (SMB) and enzymatic activity serve as sensitive indicators of soil health^[5,112]^. Wan et al.^[113]^ reported markedly increased microbial biomass carbon and microbial entropy, elevated phosphatase activities, and reduced protease activities after six years of SBFM of a natural oak forest in Xiaolongshan Nature Reserve, Gansu Province. Comparative analysis of soil quality revealed that SBFM had fewer detrimental impacts on overall soil functionality than alternative methods. Another study noted that short-term SBFM altered microbial community composition^[114]^, though the long-term effects remain unknown.

Collectively, SBFM significantly enhances soil carbon storage and elevates TN and TP content, and influences soil enzyme activity and the microbial community. However, the current research has critical limitations. Firstly, the current research focuses on carbon, nitrogen, and phosphorus while overlooking vital indicators like potassium, sulfur, and pH, which are essential for comprehensive soil fertility assessments. These studies reported that values were predominantly recorded in the topsoil (0–20 cm), neglecting the potential impacts of SBFM on deeper soil layers where nutrient dynamics and microbial processes may differ substantially. Lastly, current studies rarely account for climate seasonality, which strongly influences soil properties and microbial activity.

### Impact on stand health

Stand health arises from dynamic interactions among biotic and abiotic factors, requiring evaluation through multiple indicators^[115]^. A stand's state reflects structural and functional conditions and serves as a key indicator of the ecosystem's productivity and resilience. A recent advance is the π-value method proposed by Hui et al.^[116,117]^, which quantifies the stand's state by standardizing the ratio (*ω*) of the observed condition to an ideal optimum. Empirical studies confirm the efficacy of SBFM in enhancing stability^[45,55]^. Wan^[118]^ evaluated the stand condition of natural mixed oak-pine forest in Xiaolongshan Nature Reserve, Gansu Province, and reported a 27.09% increase in the *ω* ratio within managed stands after five years of SBFM treatment, which changed the stand's state from "poor" to "good". However, the unmanaged stands exhibited only a 13.24% increase while remaining in the "poor" category. This shows that the positive effects of SBFM diminish over time without adaptive interventions^[45]^ for maintaining long-term stability.

The π-value method represents a significant advancement in stand health assessments, but its reliance on expert-defined indicators and subjective weights introduces bias. Future refinements should focus on integrating data-driven criteria (e.g., machine learning or remote sensing metrics) to reduce subjectivity and enhance the accuracy and scalability of stability evaluations. Addressing these limitations will strengthen the applicability of such methodologies in guiding forest management practices.

### Summary

The core methodology of SBFM is to create optimal forest structure by using spatial structure parameters to guide selective cutting. This method reduces competition for resources such as light and nutrients, promoting growth of the remaining trees. This will avoid overcrowding in certain areas and reduce clustering, thereby adjusting the horizontal pattern of the stand and promoting a random distribution of the stand. Thus, SBFM adjusts the horizontal patterns toward a random distribution and decreases monospecific density while increasing species mingling. Collectively, these adjustments optimize the trees' distribution patterns, enhance species mixing, and balance size class ratios, thereby strengthening ecological stability, reducing the incidence of pests and diseases, and suppressing unfavorable microstructures.

Structural optimization further drives stand productivity. Selective removal improves the availability of resources such as light, water, and nutrients, aligning stands with natural succession patterns and enhancing growth. This process enhances the dominance of target trees, leading to a more optimal diameter distribution with fewer small trees and a greater abundance of medium- and large-diameter trees. Varied tree sizes and arrangements create diverse habitats and ecological niches, promoting biodiversity alongside canopy optimization, which improves the understory light environment to support species diversity and thus improving the stand's stability^[119]^.

Belowground impacts emerge through ecological cascades. SBFM optimizes forest stands' structure, indirectly influencing the soil through enhanced ecological interactions. Enhanced growth and diversity increase organic matter inputs, elevating SOC and nutrient cycling efficiency. Optimized spatial structure within a stand increases light penetration and soil temperature variability, stimulating groundcover plant growth and elevating labile carbon inputs^[120]^. This process increases labile carbon inputs to the soil, enhancing microbial activity, increasing carbon and phosphorus utilization, and leading to positive shifts in the microbial community's composition.

**Figure 7:**
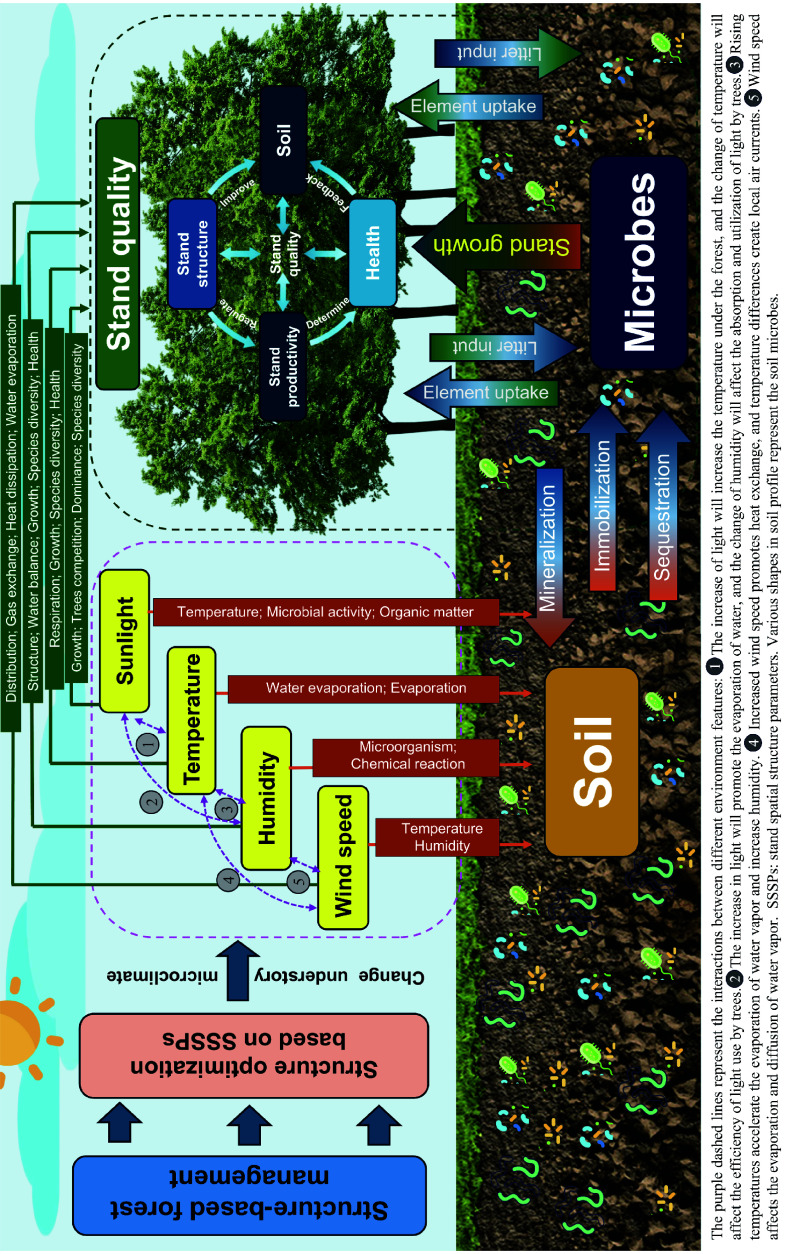
This figure illustrates the interaction mechanism of structure, environment, and function in the forest ecosystem. The core pathway is as follows: SBFM optimizes the stand's spatial structure, which regulates the understory microclimate (light, temperature, humidity, wind speed), subsequently influencing soil and microbial processes (mineralization, immobilization, sequestration, litter decomposition, etc.), ultimately affecting stand quality (structure, productivity and health), thus forming a complete ecological chain.

Structural adjustments boost productivity and the soil's functionality and health, which, in turn, reinforces overall stand quality through the interplay of aboveground and belowground ecological processes following SBFM. To aid the readers' comprehension, a diagram (Fig. 7) systematically illustrates the intrinsic mechanism through which regulation of the microclimate and soil microbial processes work in synergy to enhance the forest's ecosystem functions. This results in improved stand quality under structurally optimized conditions, guided by stand spatial structural parameters (SSSPs) via SBFM. It also elaborates on the bidirectional feedback between the soil microbial system and stand quality, as well as the interactive mechanisms among various stand quality indicators.

### Future outlook

Cross-regional long-term ecological monitoring represents a critical priority for advancing SBFM. Current research relies predominantly on the short-term (5–10 years) effects of adjusting stand structure, limiting our ability to capture the full forest succession dynamics. For instance, oil pine forests in Beijing failed to achieve an optimal spatial structure even after seven years of implementing SBFM, underscoring the need for extended temporal validation^[9]^. The core principles of SBFM have showed efficacy in specific regions, but their adaptability to diverse climatic zones (e.g., arid regions, high-altitude areas, tropical rainforests) and land-use contexts remains unclear. Future efforts should establish long-term monitoring stations in representative regions, especially arid zones and tropical rainforests, to analyze regional disparities and develop stand-type-specific management strategies.

The random structural units represent a pivotal area of ongoing research. On the basis of the uniform angle index, the five-tree structural units can be categorized as random, uniform, or clustered^[121]^. Natural forests typically exhibit more than 50% random structural units^[121]^, which is linked to the enhanced ecological stability and biodiversity observed in natural forests compared with plantations^[122]^. Therefore, creating random units in plantations by imitating natural forests is crucial for sustainable forestry development. Though research has identified plantation patterns that maximize the construction of random units^[123]^, their impacts on stand quality remain unassessed. Future studies should also explore innovative afforestation paradigms incorporating randomized planting or adaptive management.

Developing climate-resilient SBFM practices is imperative, given the escalating threats from rising temperatures, glacial retreat, sea level rise, and extreme weather events^[124]^. For example, afforestation in high-latitude regions may reduce carbon sinks' efficiency as a result of albedo effects, warranting prioritization of humid tropical zones to mitigate fire risks^[125]^. Future research should investigate SBFM strategies for restructuring stands under climate stressors like extreme droughts and wildfires. Tree species significantly affect forest soil and soil microbial elements, thereby influencing stand growth^[126]^. In addition, the forest management cycle affects multiple aspects of stands' structure, productivity, stability, biodiversity, and ecological functions^[127]^. Therefore, in the future, key study areas can include optimizing the selection of tree species and adjusting management cycles to enhance resilience under changing climatic conditions.

AI and advanced remote sensing are revolutionizing precision forest management. Innovations such as unmanned aerial vehicle (UAV) light detection and ranging (LiDAR) and hyperspectral imaging enable real-time monitoring and dynamic optimization of forest stands' spatial structures^[128]^. For example, point cloud segmentation algorithms based on UAV LiDAR (such as the PCS algorithm) can estimate the canopy cover of individual trees, with higher accuracy (*R*^2^ = 0.92)^[129]^. Deep learning models (e.g., BiLSTM) combined with multisource remote sensing datasets (e.g., SAR and LiDAR) enhance the precision of aboveground biomass estimations, addressing the limitations of traditional methods in quantifying complex uneven-aged stand structures^[130]^. In the future, realizing the combination of real-time pest and disease detection systems and intelligent logging decision algorithms will significantly improve the efficiency of management^[131]^.

Research on stand quality assessments requires further development. Over time, the concept of stand quality has shifted from single-dimensional evaluation of timber production toward a more holistic, multidimensional framework that integrates the stand's structure, health, stability, growth potential, and its ecological, economic, and social values^[15,26]^. In practice, evaluation methods such as the analytic hierarchy process (AHP)^[132]^ and comprehensive index models^[15]^ are commonly employed. The integration of advanced technologies (e.g., remote sensing and geographic information systems) has improved the efficiency and precision of data acquisition and assessments of forest management^[133,134]^. Nevertheless, several challenges persist. These include the absence of a standardized evaluation index system, difficulties in quantifying ecological and social values, unclear trade-offs in multiobjective forest management, and technical limitations in application^[133,135]^. Moving forward, research on stand quality evaluation should prioritize the development of dynamic and multiscale assessment frameworks, deepen insights into the structure–function–service nexus, and harness AI and big data technologies to enable more intelligent and precise decision-making support.

## Conclusions

This review synthesis the impact of SBFM's on stand growth, stand structure, soil properties, and stability, revealing the mechanism through which structural optimization enhances overall stand quality. By reducing competition and aligning a stand's development with natural succession, SBFM improves diameter growth, volume, and size distribution while increasing species mingling and habitat heterogeneity to support biodiversity. These aboveground improvements also strengthen belowground processes, boosting SOC, nutrient cycling efficiency, and microbial activity, forming a positive feedback loop for sustainable forest development. Future research should focus on establishing cross-regional, long-term monitoring networks to capture successional dynamics and advancing the use of random structural units in plantations to evaluate their contribution to biodiversity and stability Collectively, these advances will consolidate SBFM as a data-driven, mechanism-based paradigm for sustainable forest management under global change.

## SUPPLEMENTARY DATA

Supplementary data to this article can be found online.

## Data Availability

All data generated or analyzed during this study are included in this published article.
