# Supplementary material for: Optimizing forest structure for sustainability: a review of structure-based management effects on stand quality
Source: For Res (Fayettev). 2025 Oct 29;5:e023. doi: 10.48130/forres-0025-0024 (PMC12648026; doi:10.48130/forres-0025-0024)
Supplement: Supplementary file 1 — Supplementary data to this article can be found online. [file FR-2025-5-0024-Suppl-TableS1.pdf]

Table S1. Content analysis framework for the structured literature review includes categories and subcategories

**General information**	**Methodology**	**Key indicators**	**Discussion**
Authors	Study area	Diameter at breast height	Key findings
Database	Sample size	Stand volume	Strengths
Journal	Study period	Carbon storage	Limitations
Keywords	Control group setting	Structure	Contribution
Objective		Species diversity	Future perspectives
Publisher		Soil nutrients	
Title			
Year			
